# The ‘weekend effect’ in acute medicine: a protocol for a team-based ethnography of weekend care for medical patients in acute hospital settings

**DOI:** 10.1136/bmjopen-2017-016755

**Published:** 2017-04-05

**Authors:** Carolyn Tarrant, Elizabeth Sutton, Emma Angell, Cassie P Aldridge, Amunpreet Boyal, Julian Bion

**Affiliations:** 1 University of Leicester, 22-28 Princess Road West, Leicester, LE1 6TP, UK; 2 Department of Health Sciences, University of Leicester, Leicester, UK; 3 University of Birmingham, Birmingham, UK; 4 Primary Care Clinical Sciences, University of Birmingham, Birmingham, UK

**Keywords:** ACCIDENT & EMERGENCY MEDICINE, QUALITATIVE RESEARCH, Weekend effect

## Abstract

**Introduction:**

It is now well-recognised that patients admitted to hospital on weekends are at higher risk of death than those admitted during weekdays. However, the causes of this ‘weekend effect’ are poorly understood. Some contend that there is a deficit of medical staff on weekends resulting in poorer quality care, whereas others find that patients admitted to hospital on weekends are sicker and therefore at higher risk of adverse outcomes. Clarifying the causal pathway is clearly important in order to identify effective solutions. In this article we describe an ethnographic approach to evaluating the organisation and delivery of medical care on weekends compared with weekdays, with a specific focus on the role of medical staff as part of National Health Service England’s plan to implement 7-day services.

**Methods and analysis:**

We will conduct an ethnographic study of 20 acute hospitals in England between April 2016 and March 2018 as part of the High-intensity Specialist-Led Acute Care project (www.hislac.org). Data will be collected through observations and shadowing, and interviews with staff, in 10 hospitals with higher intensity specialist (consultant) staffing on weekends and 10 with lower intensity specialist staffing. Interviews will be conducted with up to 20 patients sampled from two high-intensity and two low-intensity sites. We will coordinate, compare and contrast observations across our team of ethnographers. Analysis will be both in-depth and cross-cutting, exploring specific features within individual sites and making comparisons between them. We outline how data collection and analysis will be facilitated and organised.

**Ethics and dissemination:**

The project has received ethics approval from the South West Wales Research Ethics Committee: Reference 13/WA/0372. Informed consent will be obtained for all interview participants. The findings will be disseminated through peer-reviewed publications in high-quality journals and at national and international conferences.

**Strengths and limitations of this study**

The proposed ethnography offers both breadth of comparison across well-characterised case study sites and in-depth detail in the data collected.
The second round of site visits offers a fuller understanding of the way that changes do or do not occur over time.
The double analytical method provides in-depth understanding within each site, whereas the framework analysis offers a transparent and robust method of comparison.
This approach will compensate for potential diversity and complexity of findings between sites, the relative brevity of the sampling frame and the possible changes in research staff during a longitudinal study.
Variation between sites in implementing 7-day standards will be captured as ‘snapshots’ rather than as a continuous analysis.

## Introduction

### The ‘weekend effect’ in hospitals

The association of weekend admission to hospital with higher death rates is a widely reported phenomenon. Studies from Canada,^1^ the USA,^2^ the UK^3–5^ and Australia^6^ show that there is an increased risk of death for patients admitted to the hospital on weekends, compared with those admitted on weekdays. Significant uncertainty exists about the causes of the weekend effect.^7^ One study reports higher error rates on weekends,^8^ whereas others suggest that the weekend effect could be a data artefact or reflect a different case mix on weekends.^1 9–11^ The organisation of care on weekends may be a factor in increased mortality; nurse staffing levels have been found to be associated with mortality, but the association between specialist intensity and mortality is less clear.^1 12^ Care quality also varies from the week to the weekend.^13^ There is currently considerable political pressure to increase 7-day services,^14^ with a focus on increasing the intensity of specialist input in hospitals on the weekend; this political imperative is, however, being driven forward without a full understanding of why the weekend effect exists, or the key ways in which care is organised and delivered differently on weekends compared with weekdays.^15^ To unravel the complexities of the weekend effect, the High-intensity Specialist-Led Acute Care (HiSLAC) study was funded by the Health Services and Delivery Research (HS&DR) programme of National Institute for Health Research (NIHR).

### The HiSLAC study

This 5-year national mixed-methods study aims to determine whether the presence of more specialists is associated with better outcomes for patients admitted to English National Health Service (NHS) hospitals on weekends. The methodology includes multiple components: a systematic literature review,^16^ surveys, case record reviews, patient outcomes analyses and ethnography (http://www.hislac.org/). A baseline measurement of specialist intensity through a point prevalence survey of hospital trusts in England receiving unselected emergency admissions revealed that the median specialist intensity on Sunday was only 48% of that on Wednesday. However no significant association was found between Sunday-to-Wednesday specialist intensity ratios and weekend-to-weekday mortality.^17^ The second phase of the study examines weekend-weekday differences in greater detail in a subset of 20 hospitals, with parallel ethnographic observations in the workplace. The 20 hospital sites will be purposively selected based on size and level of specialist intensity, to include 10 sites with relatively high levels of specialist input on weekends (HiSLAC) and 10 with relatively low levels of weekend specialist input (LoSLAC); specialist intensity is determined from the national point prevalence survey.^17^ We describe here the ethnographic component of the HiSLAC study.

The ethnographic component focuses on 20 hospitals to explore in more depth approaches to providing specialist input to medical patients, and other key features of the organisation, delivery and experiences of medical care on weekends compared with weekdays. The study will be a focused, team-based ethnography involving non-participant observations and interviews.

### A focused, team-based ethnography

Ethnography involves researchers collecting relatively unstructured data based on observations and informal conversations ‘in the field’.^18^ Ethnography has been described as ‘the art and science of describing a group or culture’^19^ and is particularly suited to social policy research because it is an approach that comes ‘closest to the people being studied’, offering opportunities to highlight what the implications of policies are on those on the ground.^20^ Ethnography enables the exploration of collective culture, that is the practices, beliefs and knowledge^21^ that make up ‘what people know, believe and do’^22^ and aims to query ‘understandings and practices that are taken for granted’.^23^ It involves exploring how context shapes and influences events and practices.^24 25^

Conducting ethnography in 20 fieldwork sites requires an approach that delimits the field of study^26^ and narrows down the object of that enquiry. A focused ethnography ‘usually deals with a distinct problem in a specific context’.^21^ In the traditional lone researcher style of ethnography, researchers spend significant periods of time ‘hanging out’^27^ with key informants, securing trust and acceptance, and taking time to let themes emerge. Focused ethnography involves conducting fieldwork over a shorter time period, with the lines and field of enquiry being clearly stated from the outset. This focused approach is akin to taking an ‘intensive journey to knowing’.^28^ Our study takes this latter approach.

Over the last decade there has been a shift from the traditional one-researcher model of ethnography to the increased use of team-based, collaborative ethnography.^29^ Our multisited study will be employing a team-based approach. Partly pragmatic, as it would not be feasible for one ethnographer to conduct all 20 fieldwork sites over a constrained time period, it also provides an alternative model that enables a more collaborative understanding of phenomena to be reached through the pooling of ideas and hypotheses throughout the research process,^30^ arguably generating a deeper and richer collective understanding.^29^ Although some contend that teamwork produces ‘rich, trustworthy ethnography’,^31^ the same authors have also highlighted numerous pitfalls, particularly if team members do not trust each other.^31^ As such, team-based ethnography involves relationship-building, coordination and collaboration between researchers in generating and interpreting data.^32^ This protocol provides a detailed account of how we intend to organise and manage the fieldwork and the data thus generated in a project of such a large scale.

### Aims, research questions and study design

The study aims to characterise the features of weekend care for acute medical patients in 10 HiSLAC and 10 LoSLAC hospitals, and explore how the organisation and delivery of specialist input on weekends vary between hospitals. We refer to specialists (rather than consultants) to mean any doctor who has successfully obtained a Certificate of Completion of Training (or equivalent). Acute medical patients are defined as medical, that is non-operative, adult patients admitted to the hospital through an emergency pathway (emergency department, acute medical units (AMUs) or similar). The study will investigate the impact of intensity of specialist input on patient care process and outcomes, and the mechanisms through which specialist intensity has an impact. The study will also explore the wider social and contextual influences on the organisation and delivery of weekend care. The findings from the ethnographic study will inform interpretation of the quantitative data collected as part of an in-depth study of 20 hospitals.

The following are the research questions: 

How do hospitals organise their specialist staffing and review of medical patients on weekends as compared with weekdays?
What is the impact of different models of specialist staffing on weekends on care delivery, patient and staff experience, and patient outcomes?
How have hospitals responded to policies for increased specialist intensity on weekends, and what challenges have they faced?
What are the other key features of the organisation and delivery of weekend (as opposed to weekday) care to acute medical admission patients, and how do these impact on patient and staff experience, and patient outcomes?
How do contextual and social factors underpin variations in delivery of weekend care to acute medical patients?

### Methods and analysis

#### Fieldwork

Ethnographic fieldwork will be conducted in all 20 hospital sites. This will involve two visits to each site; the first round of fieldwork will be conducted between April 2016 and April 2017, and the second round between September 2017 and March 2018. Two high-intensity trusts (one large, one small) and two low-intensity trusts (one large, one small) will be selected from the 20 case study sites for more in-depth fieldwork.

The first round of fieldwork will focus on characterising the roles and working practices of specialists on weekends in each hospital, as well as identifying other key weekend/weekday differences and their impact on patient care; the focus of the second round of fieldwork will be informed by findings from the first round, but will include a focus on the implementation of 7-day standards and changes in specialist intensity over time. Researchers will also conduct telephone interviews with each site’s local project lead at the midpoint between the fieldwork visits.

The qualitative lead (CT) and ES will conduct one pilot visit to a site before starting fieldwork. This pilot visit will provide an opportunity for the non-clinical ethnography team to become familiar with the acute care settings, to develop approaches for the fieldwork and to identify the key stakeholder roles for interviews. It will also inform the development of a structured observation guide and interview topic guides for interviews. In planning the fieldwork, we will also draw on two focus groups, one with clinicians and one with patients and carers.^16^

#### Observations

Observation visits will be conducted over a minimum of 4 days between Thursday afternoon and Tuesday evening, thereby capturing both weekday and weekend experiences. A range of medical acute admitting wards will be included, with observations being made along the pathway of acute medical patients admitted to the hospital, including emergency departments, AMUs, medical wards and intensive care units. Researchers will observe practices within each setting and will shadow staff including specialists and junior doctors. In each site, an on-call doctor providing cover for acute medical admissions over the weekend will be shadowed at some point during the visit. The data collected will consist of field notes from observations and informal chats with hospital staff, patients and relatives.

A structured observation guide for the first round of fieldwork visits has been developed based on the pilot fieldwork visit (see online supplementary appendix 1). This details the aims of the observations and the topics and issues on which data should be collected during observations. Researchers will focus on observing weekend staffing levels and how staffing is managed, the functioning of ward teams and other teams that support specialist-delivered care, and the nature of formal and informal reviews and handovers for acute medical patients. Researchers will also aim to collect data on salient features of the local systems, social factors and organisational context that may impact on efforts to increase specialist intensity and other aspects of 7-day services on the weekend. A structured observation guide for the second round of fieldwork visits will be developed based on initial analysis of data from the first round of visits.

10.1136/bmjopen-2017-016755.supp1 supplementary data

#### Interviews with hospital staff

Semistructured interviews will be conducted with three to five members of staff involved in providing care to acute medical patients on weekends (including those in a range of clinical roles) at each fieldwork visit. Potential participants will be identified by the local lead and through contacts made during fieldwork. Face-to-face interviews will be conducted during site visits where possible; telephone interviews will be arranged with staff who are not available during the visit or who would prefer a telephone interview. Staff interviews will be conducted using a topic guide based on findings from the pilot fieldwork visit and the pilot focus groups (see online supplementary appendix 2). Each interview will be tailored to the individual staff member’s role and will also explore issues that arise during observations

10.1136/bmjopen-2017-016755.supp2 supplementary data

#### Interviews with patients and relatives

We will interview up to 20 patients/relatives in each round of fieldwork, sampled from the four in-depth sites (two HiSLAC and two LoSLAC). Healthcare teams in participating hospitals will be asked to identify patients/relatives who are being cared for as acute medical patients during the fieldwork visit and who might be suitable candidates for interview (patients/relatives who are not too unwell or distressed to be approached). Suitable patients and/or their relatives will be approached by a member of the patient’s healthcare team to ask if they are interested in talking to a researcher about taking part. Patients will only be approached for interview if they are considered by their healthcare team to be well enough and to have the capacity; they will be offered the opportunity to be interviewed in a private space, or at a later date, for example following discharge, in which the researcher will ask for informed consent to the interview and to the retention of contact details. Participants will be informed that they can opt out of being interviewed at a later stage if they so wish. Interviews will be conducted using a topic guide developed through pilot work (see online supplementary appendix 3).

10.1136/bmjopen-2017-016755.supp3 supplementary data

#### Interviews with project leads

The telephone interviews with project leads at each site will take place in between the first and second rounds of fieldwork and will focus on exploring the issues identified during the first round of fieldwork visits. Specifically, topics will include the organisation of medical cover for acute medical patients over the weekend, how medical patients admitted on the weekend are reviewed, the pathways of medical patients admitted to the hospital and implementation of increased specialist intensity on the weekend and other elements of 7-day services.

### Facilitating a team-based ethnographic approach

Before the first observation visit at each site, we will conduct an initial set-up meeting with the local project lead (a clinician involved in acute care) to introduce the qualitative team, outline our aims and coordinate plans for the initial ethnographic visit.

A team of six researchers will be allocated to conduct observations across sites, with each site receiving one ethnographer. The study ethnographic researchers will be experienced qualitative researchers with training in research practice and equipped with research passports. A half-day briefing session will be held for the researchers; this will include a project overview, followed by a focus on the aims of the ethnography, a review of the structured observation guide and a discussion of coordination of approaches to fieldwork at each site. Researchers will also be individually briefed by the qualitative lead before their first fieldwork visit. The use of a structured observation guide will enable researchers to have an a priori framework that will help bound the field and focus their enquiry in each of the 20 very different sites. All researchers will be blinded as to which sites are of high intensity or low intensity.

The ethnography researcher team will be supported by a fieldwork project manager who will coordinate observational visits, manage data, and ensure ethical and governance requirements are adhered to. The fieldwork periods will be negotiated by the fieldwork project manager who will liaise with each site’s local project lead following confirmation of site governance approval. The local project lead will act as a collaborator by introducing the ethnography researcher to key members of staff and facilitating access to medical wards. Potential dates for conducting the fieldwork will be initially agreed with the site project lead; we will aim to identify weekends for fieldwork visits which align with the local lead’s rota, so that the lead will be on site when the researcher arrives in order to facilitate access. The fieldwork manager will arrange letters of access, keep an up-to-date log of researcher availability, allocate a researcher to the site and coordinate the visit with the local lead. All researchers will be blinded as to which sites are high-intensity or low-intensity in order to avoid bias.

To facilitate ethnography, each researcher will be provided with a pack before starting fieldwork. This will contain a checklist (see online supplementary COREQ Checklist) (to ensure that they carry with them their travel and hotel documentation, their recorder, notebook, etc), site contextual information (eg, maps, diagrams), site address and contacts, a file-naming convention to ensure correct anonymisation and labelling of documentation and audio files, and a provisional fieldwork timetable as agreed with the local lead. It will also include copies of all the required fieldwork documentation: consent forms, interview topic guides and observation guides, information sheets, and posters. We will operate a system for researcher safety, which will involve each researcher confirming by text to the fieldwork manager that he or she has arrived at the site safely, and texting again once he or she returns home.

10.1136/bmjopen-2017-016755.supp4 supplementary data

**Figure 1:**
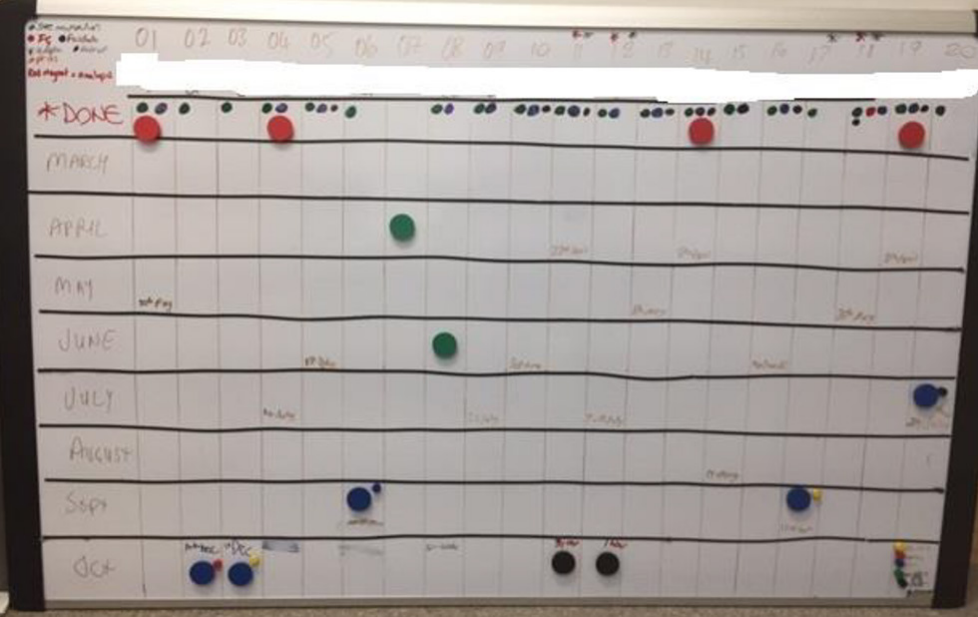
The ethnography operational whiteboard.

The fieldwork project manager will maintain an up-to-date overview of all of the fieldwork sites, logging completion of key tasks (eg, site initiation visits, governance approvals, fieldwork dates) and recording which researcher is responsible for conducting the ethnography in each site and when they will be there. The team will use an operational whiteboard detailing the status of the ethnography across all the sites (illustrated in figure 1). The fieldwork project manager will keep a record of data collection at each site (eg, number of hours of observations and location of observations within the hospital, number of interviews, job roles of interviewees, etc). Each researcher will be responsible for labelling his or her recordings as per a standard file-naming convention. Recordings will be sent to the fieldwork manager to send to a transcription company for transcribing. The fieldwork manager will keep track of the status of each piece of data, for example, dates sent to transcriber, whether the transcript has been returned, whether it has been imported into NVivo (qualitative analysis software) and whether it has been coded.

### The process of capturing observational data

#### Individual accounts

At the end of each day in the field, the researchers will use an encrypted audio recorder to convert their written field notes to a recording of their observations, who they had seen and their reflections on what was happening on weekends compared with weekdays. Audio recording the ethnographers’ experiences this way will act as the first step on the analytic pathway, as researchers reflect on what they have seen, and begin to interpret their observations and challenge their assumptions. The researchers’ reflections act as a way of translating the field, as they move from making observations to analysis while describing events and processes at each site.

#### Collective accounts

Regular debriefing sessions will be held to enable a group of researchers to come together to discuss three to four fieldwork visits. These sessions will help ensure that the data collection remains focused on core topics, and that emerging themes are explored and used to inform subsequent data collection. The fieldwork project manager will bring together those researchers who have recently completed their time in the field as soon as possible afterwards. The team debriefs will include the qualitative lead (CT), who will take the lead in asking questions of the other researchers.^33^ Debriefs will be structured in a similar format to those laid out by Schoepfle and Werner consisting both of overarching or ‘grand tour’ questions and ‘mini tour’, or more detailed questions that will encourage the flow of both contextual and in-depth information. The researchers will collectively talk through their field notes and describe their interpretations of what happened at their fieldwork site(s). During each session, which will last up to 3 hours, each ethnographer will recount his or her stories from the field in a way that he or she is most comfortable with,^33^ but will be encouraged to be open to questions from other team members in order to ‘co-produce accurate descriptions rather than test the memory of another participant’.^33^ Coproducing descriptions therefore means trusting other researchers within the team to be able to share and speak openly without fear of censure.^29^ This approach, argues Scales, results in researchers having to be collaborative in nature and having to ‘share their observations, and confront inconsistencies between interpretations’ (p. 24).^29^

In essence, the debrief will act as an interview about the interpretation of all the observations across a number of sites. It will enable us to capture contextual information that may well be otherwise missed^33^ and allow researchers to reflect on their interpretations in the light of other researchers’ perspectives and understandings, thus incorporating reflexivity into the collective process.^34^ They will enable us to explore the asymmetries that can arise in conducting team ethnography,^32^ providing checks and counterbalances to different understandings of events. All the sessions will be audio-recorded. Transcripts of debriefs will be included in the analytical process, acting as a bridge between individual accounts and collective interpretation of data.

### Analysis

Interviews, field notes and debriefs will be transcribed verbatim and imported into NVivo for coding and analysis. Analysis will draw on elements of grounded theory, in particular the constant comparative approach,^35 36^ and will run alongside data collection. We will develop an initial coding frame based on familiarisation through reading and line-by-line coding of a diverse selection of interview and observation transcripts, along with reflection on group debriefs. This coding frame will be used to code a small number of transcripts and be further developed to enable focused coding. We will then use this focused coding frame to code all transcripts, remaining sensitive to new emerging themes and disconfirming cases.

We will conduct two distinct elements of analysis and interpretation based on this initial coding. First, we will draw together data from each individual site to characterise the features of weekend care in each of the 20 hospitals. We will use framework matrices in NVivo to chart the key features of each individual hospital across all 20 sites. This method enables us to group individual transcripts from each site into cases. Each case will then be charted into cells under each of the themes emerging from the first stage of analysis. The summaries in the cells of the charts will thus be linked to and grounded in the ‘raw’ qualitative data in NVivo, thus making the process of cross-case comparison transparent and robust.^37^ Supported by this process we will produce a narrative case study of each site. These case studies will describe how weekend care is organised and delivered for acute medical patients and provide key contextual information about each site. The case studies, along with the more detailed matrices, will be used to characterise features of weekend care in each site and to inform the interpretation of the quantitative findings from the wider HiSLAC study. This approach will help us manage the large volumes of data generated across multiple sites.

Second, we will conduct a grounded analysis of cross-cutting themes across sites. We will flesh out concepts by moving between debriefs and the observation and interview data, linking the grounded data in the transcripts to the more analytical constructs discussed in the debriefs, and reflecting on tensions and synergies between etic and emic accounts.^38^ This process will enable us to build on the multiple contributions of the ethnography research team to interpreting the data. We will produce data summaries across sites organised by emerging themes, based on initial coding, as a means of interpreting the data.

Analysis will be led by CT and ES; the ethnography team will be involved in data interpretation, through sharing of emergent findings, case studies and theme summaries with them for discussion and feedback.

### Ethics and dissemination

We have obtained a favourable ethical approval from the Proportionate Review Sub-committee of the South West Wales Research Ethics Committee: Reference 13/WAS/0372, 12 November 2013.

During our fieldwork we will make every endeavour to be respectful and mindful of patients’ privacy, dignity and rights, and will negotiate access to situations for observations sensitively and with as little impact as possible on the situations we are observing. We will take care to ensure that we do not distract staff from their clinical duties.

All participating sites will be informed about the periods of fieldwork; posters detailing the study and including a photograph of the allocated researcher will be displayed in relevant wards and areas. A communications associate will also make contact with each trust’s communications department approximately a week in advance of the field visit. Potential interview participants will receive written information sheets outlining the study and its purpose, and informed consent will be obtained from staff and patients prior to interview. Consent forms will be retained by the local project lead at each hospital site, as stipulated by the ethics committee, and will be stored securely. All interview and observation recordings will be given a code number and will be anonymised during transcription. We will use transcribing companies with which we have established relationships and existing confidentiality agreements. Findings from the study will be published in peer-reviewed journals and presented at relevant conferences; findings will also be made publicly available on the HiSLAC study website (www.hislac.org).

## Conclusion

Finding out how services for acute medical patients are organised across 20 different hospital sites is challenging and complex. There is a clear need to constrain the focus of the study in each site in order to produce detailed and comparable findings, as well as to complete the fieldwork on time. The scale of the study requires well-targeted research questions, focusing on how staffing is organised and care is delivered on weekends compared with weekdays. The interviews with patients and staff add further context to enable us to understand more of what happens on the ground within each site. The study requires continuous flexibility, both in the planning of the fieldwork and also in conducting it in situ. A study of this size demands a high level of project management to organise and oversee the data collection, and requires collaboration and trust between the researchers taking part. Researcher debriefs play a crucial part in choreographing the volume of observations and managing the analytic process. They help manage the data, explore patterns across the sites and move from description to interpretation. The findings will make a significant contribution to understanding the weekend effect in acute medical care, and the impact of specialist staffing on weekends on care delivery, patient and staff experience, and patient outcomes.

## Supplementary Material

Reviewer comments
