# Supplementary material for: The ‘weekend effect’ in acute medicine: a protocol for a team-based ethnography of weekend care for medical patients in acute hospital settings
Source: BMJ Open. 2017 Apr 5;7(4):e016755. doi: 10.1136/bmjopen-2017-016755 (PMC5719649; doi:10.1136/bmjopen-2017-016755)
Supplement: supplementary data [file bmjopen-2017-016755supp001.pdf]

ccp3@le.ac.ukAppendix 1

Observation guide, HiSLAC ethnography round 1

Aims

To describe how each theme covered below affects the operation of the hospital at weekends and **how it differs from weekdays**, and describe the impact that it may have for patients, with a focus on **urgent and emergency medical patients.**

Themes:

Staffing levels

- In each area visited, explore how senior doctor staffing is coordinated over the weekend and how this compares to weekdays *Look for: numbers and specialties of senior doctors who are available; whether doctors are working in blocks of multiple days*
- In each area visited, explore levels of staffing across all roles and grades – senior, mid-grade, junior doctors, nurses, HCAs and admin e.g. ward clerks; numbers of agency and locum staff; whether there are skills shortages at weekends.

ED/CDU/AMU - case mix and demand

- Are the demographics of medical patients being admitted at weekends different to the week? In what way, and why? What impact does this have?
- What particular pressures does the hospital face at the weekend (e.g. demand on ED)?

CDU/AMU - patient review

- Describe how consultant review is co-ordinated in CDU/AMU.
  - *who reviews medical patients – which speciality are they from? How soon do patients get reviewed by a consultant once admitted to CDU? How often? Is this different at weekends to weekdays?*
- What happens when patients are moved from CDU/AMU on to a ward? Are arrangements made to make sure they are reviewed within 24 hours?

Medical wards –medical cover

- What form does consultant input take at weekends? Is this different to weekdays?
  - *Are consultants on or off site?*
  - *Are consultants covering the area they usually cover during the week or is this different?*
  - *Is the consultant "passing through" or do they remain on the ward for a set time period?*
- How is junior doctor input organised over the weekend? Are there 'on call doctors' – what areas do they cover? What are on call/junior doctors doing at the weekend?

Medical wards – (specialist) patient review

- Describe arrangements for specialist review of patients on medical wards over the weekends, what form review takes (e.g. consultant led ward rounds), and how frequently it happens
  - *Who reviews these patients*
    - *consultant, SHO…*
    - *are patients reviewed by doctors from the matched speciality (e.g. respiratory patients by respiratory consultants)?*
  - *How is the system for review managed*

- *is it proactive i.e. is there an active list of patients each day for review, or are patients just reviewed reactively e.g. if they deteriorate*
- *is it based on inclusion (see only those identified as requiring review) or exclusion (i.e see all patients unless identified as not requiring review)*

  - *How are decisions made about who is reviewed and who is not reviewed? Who makes these decisions? What are the prompts for patient review at the weekend e.g.*
    - *newly admitted,*
    - *unstable / uncertain management plan*
    - *outstanding tasks*
    - *concerned relatives,*
    - *potential discharge*

- Is the system for review different across specialities?
- How does patient review at the weekend compare to weekday?

Specialist input

- What exactly are specialists doing, and what difference does specialist input (or <u>lack of specialist input</u>) make? Collect examples of this e.g. spotting missed diagnosis, expediting tests.
- How are consultant decisions actioned at the weekend? Do any problems arise?
- How easy is it for junior doctors / nurses to get input and advice from a more senior doctor, or from a specific specialty at the weekend? Do junior doctors seem comfortable seeking senior support/input?

Handovers and communication

- Describe how handovers happen at weekend, and from the weekend to the weekday. Is this different from weekday handovers?

  Who's involved – are consultants involved in weekend handovers? Where do they take place, how are they structured and what is handed over.
- Describe quality of communication and multi-disciplinary working

  Are patient records on paper or electronic, and what electronic systems do they have e.g. e-prescribing?

  What is communication between doctors and nurses like? Is there evidence of multidisciplinary working at weekends?

Deteriorating patients and urgency

- How are deteriorating patients detected and responded to over weekends?

  Look for /ask : What happens if staff become worried about a patient? How does the local Early Warning Score system (EWS) work? Is it a paper or electronic system (are there automatic flags); who is responsible for responding e.g. outreach team)? How well does the system work? Problems with transfers to ICU at the weekend?
- Probe for staff understanding of 'emergency / urgent / non-urgent' in relation to requests for consultant review and for diagnostic tests / therapy. Do delays at weekends for less urgent things really matter? Why?

Access to diagnostics, therapy, and other services

- Describe issues that arise relating to availability of services at weekends as compared to weekdays:
  - *diagnostic services e.g. x-ray, cardio testing, CT, blood tests and results (does this differ for urgent cases and for less urgent cases?)*
  - *therapy services and allied health professionals e.g. physio, OT, nutrition, SALT, microbiologist, drug and alcohol services, mental health services*
  - *pharmacy for ordering medication / pharmacist input e.g. meds recs*
  - *resources or systems to support discharge at the weekend e.g. do they have a discharge team, access to community services (at weekend)*?
  - *access to palliative care at weekends both within and outside the hospital?*

Safety and quality of care

- Is there evidence of risks to the quality or safety of patient management or care due to problems at the weekend?
    - *evidence of differences in tempo and flow at weekends, evidence of delays in diagnosis, or treatment, problems with patient flow*
    - *problems that rise around transitions e.g. between AMU&wards, into ITU, discharge*
    - *problems due to patient deterioration; or other problems with patient care arising because of the nature of weekend working*
- Is there evidence of a weekend work ethic, or problems being taken for granted because it's the weekend? Is there evidence of staff informally compensating for or working round problems that arise over the weekend?
- Any evidence of systematic safety-focused activities (e.g. advance planning for the weekend / safety briefs / safety huddles)?
- How do staff approach management on Mondays (e.g. do they prioritise the sickest patients first on ward rounds)?

Patient Experience

- Is there evidence of staff taking time to talk to patients, keeping them informed, involving in decisions? Is this different at the weekend to weekdays?
- Researcher to chat informally to patients and relatives about:
    - *How has it been over the weekend in hospital?*
    - *Has a doctor been to see you? Do you know which speciality they are from and whether they are a consultant, junior etc*
    - *How much have staff talked to you about what's going on?*
    - *Have you had enough chance to ask questions?*
    - *How quickly have things been sorted out for you, and have there been any delays or problems?*
    - *Is there anything you've felt worried or unhappy about?*
    - *How secure and confident have you felt during your stay at the weekend?*
