# Supplementary material for: The ‘weekend effect’ in acute medicine: a protocol for a team-based ethnography of weekend care for medical patients in acute hospital settings
Source: BMJ Open. 2017 Apr 5;7(4):e016755. doi: 10.1136/bmjopen-2017-016755 (PMC5719649; doi:10.1136/bmjopen-2017-016755)
Supplement: supplementary data [file bmjopen-2017-016755supp002.pdf]

Appendix 2

HiSLAC staff topic guide (to be tailored to role)

Introduction

This project is looking at the differences in the way hospitals work on weekends and weekdays, and the extent to which different staffing models make a difference in reducing the weekend effect. We would like to ask you for your views and experiences of weekend care organisation and delivery in comparison to weekdays, and what effect this may have on staff and patients. We would particularly like to focus on the treatment and care of **urgent and emergency medical patients**. *There are no right or wrong answers - we are interested in what you think and what you have experienced. Everything you tell us remains within the research team and will be anonymised. Fill in consent form Remind about recording and check all ok.*

Opening

- Can you tell me a bit about you and your role here? What types of patients do you work with?
- How long have you worked here?
- What is your normal pattern of working?

Weekend and weekday differences

What is it like for you working at the weekends? Is this different to weekdays? In what way?

Do you face any challenges over the weekend? Probe for staffing levels, specialist intensity, availability of diagnostic, allied, therapy pharmacy services, problems with patient flow etc. Why do these matter?

Do you think there are any differences in the case mix of the medical patients admitted at weekends as compared to weekdays? What differences are there and why? What are the implication of this?

When medical patients are admitted as an emergency at the weekend, as compared to a weekday, what problems can arise? Probe – deterioration, delays in tests etc.? *Can you give any examples of patient cases to illustrate*

Are there any particular risks for emergency medical patients who are admitted at the weekend at this hospital as compared to in the weekday?

Are there any differences in terms of communication with patients and their relatives, and patient involvement, when it's the weekend? Why?

Is there anything you think should change to improve the treatment and care of emergency medical patients who are admitted over the weekend?

Is there anything you think already works particularly well here at the weekend? What is this, and why?

Specialist intensity

Can you tell me more about the consultants and their role at weekends - What is the level of consultant availability at the weekend (*specify area e.g. this ward / these general medical wards*)? How many consultants are available and where are they based? What do they do at the weekend? Does this differ to weekdays? *(probe for patient review/ availability for decision-making / handovers etc).*

How is review of emergency medical patients coordinated at the weekend (e.g. regular ward rounds); who reviews patients? Who gets reviewed and how is this decided?

How easy is it to get consultant support at weekends (e.g. if you are worried about a patient or need advice or a decision making)? Do people feel comfortable to approach or phone consultants for advice at the weekend? To what extent do you (or jnr doctors/trainees) feel supported in decision-making at weekends? *Can you give any examples of when you felt this was a problem, or when this worked well?*

What difference does having specialist input (or lack of specialist input) at the weekend make for patient treatment or progress – what do specialists do that makes a difference? *Can you give any examples based on patient cases?*

Increased specialist working

To what extent are you aware of the move to increase specialist/consultant presence in the hospital at the weekends? How do you feel about this initiative?

Do you think this will help improve outcomes for emergency medical patients admitted at weekends? Why/ why not?

How does having increased specialist input at weekends make a difference to patient treatment?

- *Probe for: spotting missed diagnosis, amending treatment, giving sound advice, carrying out emergency procedures, another pair of hands*

Do you think that just increasing consultant presence is enough to improve outcomes for patients admitted at weekends? Or do other things need to happen too in order to realise a benefit? What and why?

What would need to happen to achieve increased consultant presence at weekends here? Do you think there are any barriers or challenges in making it work? (probe for financial, resistance from consultants…).

Do you foresee any negative consequences? How might these be managed?

Closing

Thank you for talking to me today, is there anything else you would like to add about how care is organised and delivered here at weekends?
