# Supplementary material for: The ‘weekend effect’ in acute medicine: a protocol for a team-based ethnography of weekend care for medical patients in acute hospital settings
Source: BMJ Open. 2017 Apr 5;7(4):e016755. doi: 10.1136/bmjopen-2017-016755 (PMC5719649; doi:10.1136/bmjopen-2017-016755)
Supplement: supplementary data [file bmjopen-2017-016755supp003.pdf]

Appendix 3

HiSLAC Patient topic guide

Patient/relative interviews will explore their experiences of care in the hospital on week days and weekends: the extent to which care was prompt, attentive, and met their needs; how easy it was to get their questions answered; how often they saw a doctor, whether they saw junior or senior doctors, and whether this was something they are aware of/concerned about. They will also be asked about their overall views of the quality and safety of the hospital.

Introduction

Thank you for taking the time to talk to me today. My name is… I'm part of a research project that is investigating how care is organised, delivered and experienced in 20 different hospitals in England. We are particularly interested in finding out if your experience of care and treatment differs at the weekend compared to weekdays. I'm interested in finding out your views and experiences of the care you received during your most recent stay in hospital. There are no right or wrong answers and we welcome your thoughts, views and experiences. Everything that you tell me will remain confidential within our research team. We do not attach your name to any documentation and your views will remain anonymous.

Check ok to talk, ok with recording, sign consent form (& ensure stored securely at the trust following the interview)

Opening

Could you tell me about your/your relative's most recent hospital admission – can you tell me what happened?

Check understanding of route taken to end up as emergency admission and which day events happened on throughout.

Take a narrative approach to get the patient / relative to describe their experience and what mattered to them, but use the questions below AS PROMPTS as needed to flesh out story

ED

What happened in the ED. How long were you/relative there?

Tell me about what happened after you/your relative had been seen in the emergency department (if relevant). *Probe for where they went to next*

Admission

When you were in [CDU/AMU]…

- How quickly were you/relative seen by a doctor?
- Did the doctor introduce themselves? Probe for whether junior or senior, from which speciality? Was this who you expected to see? If not why not?

- Can you remember what they did to start with? *Probe for – whether sent for tests etc/blood tests*

Were there any problems or delays in getting tests or getting other input (e.g. a specialist doctor to review you) at this stage?

Did you/relative see a doctor more than once on CDU? When did you see them? What did they do?

Did you feel as though you were kept up-to-date with what was happening?

What happened next and how was this decided?

Ongoing treatment and care/ on the ward

When you/relative were on the ward…

- Did you/they see doctor(s) over the weekend while you/they were on the ward? *At what points did you see them and what did they do?*
- Did the doctor(s) introduce themselves? Do you know if you/they saw a consultant? P*robe for whether junior or senior, from which speciality? Was this who you expected to see?* If not why not?
- Were you happy with the level of input from the doctors over the weekend?

What happened in making decisions your/your relative's treatment while you/they were on the ward? Were there any problems or delays?

Were tests / investigations (eg CT) done / medication sorted out / treatment started / therapy and service organised? Were there any delays in getting these things organised?

Were there any other problems for you/your relative over the weekend? *Probe for getting therapy pain relief / dignity / support with using toilet etc*

To what extent did you/your relative feel you could get any questions answered? Did you feel you were you kept up to date?

Was there anything that worried you about your/relative's treatment or care over the weekend? What was this any why?

Were there differences in the treatment and care you/relative received at the weekends compared to in the week? What were these differences?

What were you told about plans for getting you/your relative home? When was this discussed, and what did staff talk to you about? Were there any problems or anything that delayed you/your relative's discharge (eg getting home assessments or other services organised at weekend)?

How long did you/they stay in hospital?

Patient safety

If I was to ask you how safe and secure you felt you were / relative was during the recent stay in hospital – what do you understand by this? *Probe: What does feeling safe in hospital consist of?*

So how safe and secure did you feel you were / relative was during the whole stay? At the weekend / in the week? Why?

What in particular made you feel it was safe?

What in particular made you feel it was unsafe or made you anxious? Why? Were you able to do anything about this?

What do you think the patient's / relative's role is in making care safer in hospital?

Probe – some staff think that patients / relatives

a) *should play a role for example by keeping an eye on their medicines and ask staff to wash their hands. What do you think about this? How do you feel about that – is it something you have done or would consider? Why?*
b) *Some staff say that it is difficult for patients / relatives to raise concerns about their care. What do you think? How do you feel about that – is it something you have done or would consider? Why?*

Closing

So.. now that you have had these experiences in x hospital. Thinking back

How happy were you with how often you saw a doctor or someone in a senior position over the weekend?

What was your overall view of the treatment and care you received at the weekend at x hospital?

Is there anything about your experience of treatment and care at the weekend that you would have liked to have been different?

Is there anything you would like to add?

Thank and close.
