# Supplementary material for: The ‘weekend effect’ in acute medicine: a protocol for a team-based ethnography of weekend care for medical patients in acute hospital settings
Source: BMJ Open. 2017 Apr 5;7(4):e016755. doi: 10.1136/bmjopen-2017-016755 (PMC5719649; doi:10.1136/bmjopen-2017-016755)
Supplement: Reviewer comments [file bmjopen-2017-016755.reviewer_comments.pdf]

## PEER REVIEW HISTORY

BMJ Open publishes all reviews undertaken for accepted manuscripts. Reviewers are asked to complete a checklist review form (**http://bmjopen.bmj.com/site/about/resources/checklist.pdf**) and are provided with free text boxes to elaborate on their assessment. These free text comments are reproduced below.

## ARTICLE DETAILS

| | |
|---|---|
| **TITLE (PROVISIONAL)** | The 'weekend effect' in acute medicine: A protocol for a team-based ethnography of weekend care for medical patients in acute hospital settings |
| **AUTHORS** | Tarrant, Carolyn; Sutton, Elizabeth; Angell, Emma; Aldridge, Cassie; Bion, Julian |

This article was not externally reviewed at BMJ Open. Protocols that have been independently assessed prior to submission to BMJ Open are usually fast-tracked to publication on the grounds that further substantial changes will not be possible. This independent assessment will usually be external, independent review for both a substantial grant award from a non-commercial or government funder and ethics approval.
